# Does the Femoral Head Size in Hip Arthroplasty Influence Lower Body Movements during Squats, Gait and Stair Walking? A Clinical Pilot Study Based on Wearable Motion Sensors

**DOI:** 10.3390/s19143240

**Published:** 2019-07-23

**Authors:** Helena Grip, Kjell G Nilsson, Charlotte K Häger, Ronnie Lundström, Fredrik Öhberg

**Affiliations:** 1 Department of Radiation Sciences, Radiation Physics and Biomedical Engineering, Umeå University, SE-901 87 Umeå, Sweden; 2 Department of surgical and perioperative sciences, Umeå university, SE-901 87 Umeå, Sweden; 3 Department of Community Medicine and Rehabilitation, Physiotherapy, Umeå University, SE-901 87 Umeå, Sweden

**Keywords:** MEMS, gyroscopes, accelerometers, total hip arthroplasty, movement analysis

## Abstract

A hip prosthesis design with larger femoral head size may improve functional outcomes compared to the conventional total hip arthroplasty (THA) design. Our aim was to compare the range of motion (RoM) in lower body joints during squats, gait and stair walking using a wearable movement analysis system based on inertial measurement units (IMUs) in three age-matched male groups: 6 males with a conventional THA (THAC), 9 with a large femoral head (LFH) design, and 8 hip- and knee-asymptomatic controls (CTRL). We hypothesized that the LFH design would allow a greater hip RoM, providing movement patterns more like CTRL, and a larger side difference in hip RoM in THAC when compared to LFH and controls. IMUs were attached to the pelvis, thighs and shanks during five trials of squats, gait, and stair ascending/descending performed at self-selected speed. THAC and LFH participants completed the Hip dysfunction and Osteoarthritis Outcome Score (HOOS). The results showed a larger hip RoM during squats in LFH compared to THAC. Side differences in LFH and THAC groups (operated vs. non-operated side) indicated that movement function was not fully recovered in either group, further corroborated by non-maximal mean HOOS scores (LFH: 83 ± 13, THAC: 84 ± 19 groups, vs. normal function 100). The IMU system may have the potential to enhance clinical movement evaluations as an adjunct to clinical scales.

## 1. Introduction

Total hip arthroplasty (THA) has revolutionized treatment of arthritic hip disorders when conservative management fails to relieve pain and/or restore hip function. However, despite greatly improved hip function after THA, movement patterns may still deviate from normal, e.g., reductions in walking velocity, stride length, sagittal hip joint range of motion (RoM) and peak hip abduction compared to healthy controls [1,2,3,4]. The conventional metal-on-polyethene design is still most common since it is safe and cost-effective, but a major concern is wear of the plastic material and aseptic loosening that increasingly leads to implant failure [5]. Hence, new materials, surgical procedures and design concepts are constantly emerging [5]. Metal-on-metal prostheses were introduced, but were later removed from the market due to an even higher frequency of complications, e.g., carcinogenic effects from elevated levels of metal ions in the tissues, column fractures (especially in females with smaller-sized hips) and pronounced corrosion [6,7,8]. An interesting aspect of this design was that it allowed for a larger sized femoral head. An increased femoral head size has been reported to give a better functional outcome [9,10,11,12], but contradictory reports exist [13,14]. Lu et al. [10] compared two groups that had ceramic-on-ceramic prostheses with small versus large heads and showed that the large femoral head design allowed greater hip flexion RoM. Shrader et al. [11] found that participants with a large femoral head design (i.e., resurfacing hip arthroplasty) achieved greater hip extension during stair walking, compared to participants with conventional THA. A recent study found that the risk of revision due to dislocation is lower for people with THAs with larger head sizes, although a trade-off between stability and prosthesis survivorship was acknowledged [12]. By contrast, Petersen et al. [14] found a greater improvement in peak abductor moments during gait in the conventional THA group compared to a group with a large femoral head design. Furthermore, a study by Jensen et al. used [13] the Gait Deviation Index (GDI) to analyze gait quality after treatment with either conventional THA or a prosthesis with a large femoral head and found that the group with conventional THA improved more according to the GDI scores. Hence, evaluation of how the femoral head size affects movement function is still of great interest.

In clinical practice, hip function is commonly assessed using clinical scales such as the Hip dysfunction and Osteoarthritis Outcome Score (HOOS) [15]. However, such scales cannot offer the detailed information regarding hip function during functional activities that modern gait analysis can [16,17]. The gold standard method used for human movement analysis is stereophotogrammetry, based on 3D optical camera systems and skin markers. The major error source is skin and tissue artefacts that may affect the measured joint angles by several degrees [18,19]. Even so, optical motion capture provides important information about movement function and is commonly used for clinical gait analysis [20,21,22,23,24]. A problem with this method is that it is only available to specialized centers due to high costs and requirements of expert operation. Alternatively, portable systems based on inertial measurement units (IMUs) show promising results for different clinical applications assessing gait and lower limb joint angles [25,26,27,28,29,30,31]. A recent review compared IMU systems to standard systems used in gait analysis [31] and concluded that portable IMU systems can be used for this purpose in clinical settings with good reliability. Zugner et al. [32] evaluated the accuracy of an IMU system compared to a 3D optical camera system during gait analysis of 49 people with THA and showed that the IMU system produced valid kinematic data of lower body flexion-extension RoM, even though hip RoM may have been underestimated by a few degrees. In addition to measurements of RoM, the Gait Profile Score (GPS) and the GDI* (a log transformed and scaled version of the GPS which closely matches the GDI) are clinically interesting parameters that give an overall measure of the movement pattern during the gait [33]. These scores quantify the difference between an averaged, non-pathological gait pattern of a control group and the gait pattern of a pathological individual. The deviation is summarized into an index that indicates the absence of gait pathology [33]. Measurements of RoM, GDI* and GPS during daily activities have thus great potential to enhance the clinical evaluation of hip function as an adjunct to clinical scales.

Hence, the aim of the current study was to use a wearable IMU-based system to assess RoM, GDI* and GPS during squats, gait and stair walking in three groups: a group of asymptomatic controls, a group treated with a conventional THA (THAC) and a group treated with a large femoral head (LFH) design. We hypothesized that the LFH design would result in a greater hip RoM compared to the THA design, thus providing movement patterns more similar to asymptomatic controls. We also expected a larger side asymmetry in gait parameters for THAC compared to both LFH and controls.

## 2. Materials and Methods

### 2.1. Participants

Three groups participated: nine males with a unilateral large-sized femoral head design (LFH), six males with a unilateral conventional total hip arthroplasty (THAC), and eight controls with healthy hips and knees (CTRL), see Table 1 for patient characteristics. All patients operated with an LFH design between the years 2006–2010 were invited. Inclusion criteria were males operated at the Department of Orthopedics, University Hospital of Umeå, treated for hip arthrosis with unilateral surgery 2–6 years prior to this study. The minimum post-surgery time of 2 years was chosen to include only fully rehabilitated persons. To reduce long-term effects, the maximum post-surgery time was set to 6 years. Exclusion criterion were bilateral prosthesis, other hip disease or hip trauma. All patients that volunteered to participate and fulfilled the inclusion criteria were included in the study. The THAC Group was selected to match the LFH group regarding gender, age and activity level, based on information from patient journals. Females were excluded from this study as only males received LFH prostheses due to known complications with the LFH design related to the smaller hip size of females. The CTRL group included hip- and knee-asymptomatic, age-matched males that were recruited among hospital staff and acquaintances.

Participants of the THAC group were operated with one of two THA designs: one person received a Corail^TM^-Pinnacle^TM^ design (metal stem-ceramic head, dePuy Orthopaedics) and five persons received a Synergy^TM^-Reflection^TM^ design (metal stem-oxinimium head, Smith&Nephew). Both designs are commonly used and were assumed to be comparable (i.e., no known differences in performance exist according to Swedish Quality Registries). Participants of the LFH group were treated with one of two designs: seven persons received a resurfacing hip arthroplasty with a metal-on-metal articulation, where the femur component was cemented to the original femoral head and the cup was inserted without cement (ASR^TM^, dePuy Orthopaedics); and two persons received a Corail^TM^-ASR^TM^ design (conventional stem, large metal head, dePuy Orthopaedics). Both designs included a metal cup and a large femoral head (between 49–57 mm) and were assumed to be functionally comparable. The femoral head sizes in each group are stated in Table 1. All prostheses were inserted using a posterior surgical approach. Various surgical procedures are currently used to access the hip joint: anterior incision [34]; anterolateral/direct lateral incision [35,36,37]; and posterior incision [38,39]). Some advantages with the posterior approach are a reduced operative time, shorter hospitalization time and a shorter period of time until resumption of unprotected weight bearing [39].

All participants gave their informed consent for inclusion before they participated in the study. The study was conducted in accordance with the Declaration of Helsinki, and the protocol was approved by the Regional Ethical Review Board in Umeå, Sweden (Dnr 09-120M).

### 2.2. Measurement Protocol

The study was conducted in 2012 in a clinical setting (the Department of Orthopedics, University Hospital of Umeå, Sweden). The THAC and LFH participants first completed the HOOS score which is a validated 40-item questionnaire used to assess hip disability after total hip replacement [15]. It has five separate subscales (pain, symptoms, activities of daily living, sport and recreation function and hip-related quality of life), where each subscale is graded 0–100 (worst to best) [15].

Each participant then performed a protocol adapted from the Short Physical Performance Battery (SPPB) that assesses leg motor function [19]. One test leader (a medical student) conducted all motion registrations. Three tasks were performed in a consecutive order and took less than 30 min to perform after the IMU sensors had been attached. All tasks were conducted barefoot and consisted of 5 trials performed at self-selected speeds: (1) squats with the instruction to flex the hips and knees as much as possible without pain or discomfort; (2) nine-meter gait with analysis of four steps in the middle of the walkway; and (3) stair walking (both ascending and descending) performed in the hospital building on a staircase of 10 steps, where each step was 30.3 cm deep and 17.5 cm high. For each stair walking trial, the person started by ascending the stairs, then stopped at the top of the stairs, turned and then descended the stairs. Like the analysis of gait, only 4 steps were analyzed in the middle of the stairs at each trial. The steps at the beginning and end of each trial were removed (applies to gait and stair walking) to avoid the inclusion of accelerating and decelerating movements which occur at the beginning and end of a movement. In addition to this protocol, a series of standardized movements (pelvic flexion, hip abduction, knee abduction and knee flexion-extension) were recorded as part of a functional calibration procedure. This procedure is further described under “Motion registration and data processing”.

### 2.3. Motion Registration and Data Processing

The motions of the pelvis, hip joints and knee joints were registered with a sampling frequency of 128 Hz using a portable motion sensor system (MoLab^TM^, AnyMo AB, Umeå, Sweden). The system has been validated against a 3D optical camera system with an accuracy of about 2–3° (i.e., the mean difference in angular output) and a precision of about 2–3° (i.e., the standard deviation of the angular error) for movements of the lower body [40]. The system consisted of a battery-powered unit with a microprocessor that communicated with five IMUs including three-dimensional accelerometers and gyroscopes. The IMUs were placed on the posterior pelvis at the mid-point between the right and left spina iliaca anterior superior, on the right and left thigh 10 cm above the superior patella and on the right and left shank 10 cm below the tibial tuberosity, using elastic straps. The dynamic ranges of the 3D gyroscopes and accelerometers were ±300 degrees/s and ±10 g respectively, with a resolution of 14 bits. The IMU signals were wirelessly transmitted to a PC during the motion registration and were further analyzed in MatLab^®^ (R2018a, The MathWorks, Inc, Natick, MA, USA).

All data were lowpass filtered using a Butterworth filter with a cut-off frequency of 5 Hz prior to further calculations. Direction cosines matrices (DCM) were obtained by a quaternion-based fusion of gyroscopic and accelerometric data [41] combined with a Kalman filter that reduced drift [40]. The functional calibration recordings were used to calculate each segment’s medio-lateral and inferior-superior axes and align the coordinate frames of each portable IMU with the anatomical frame of the body segment that it was attached to. This procedure was done to ensure that the joint angles were expressed correctly [40]. The Cardan sequence XYZ was used when transforming DCMs into 3D joint angles. After alignment, +X represented flexion, +Y represented adduction and +Z represented internal rotation for the pelvis, hip and knee joints.

In total, 20 gait and stair walking cycles for each leg were selected for each respective task and participant for further analysis (i.e., 5 trials x 4 cycles). The beginning of each gait and stair walking cycle was defined as when the foot first contacted the ground (heel-down) and correspondingly, the end of each cycle was defined as the next heel-down of the same foot. Automatic identification of heel-down events was performed in MatLab as the time point of maximal hip extension in the contralateral side. Each event was then verified by visual inspection of angular curves and skeleton animations, and manually adjusted if the automatic event identification was deemed incorrect. For squats, the first trial was excluded and the last four were included in subsequent analysis. The beginning and end of each trial was defined as the point of minimal hip flexion prior to and after each squat.

Kinematic outcome measures were calculated based on pelvis, hip and knee joint angles (flexion-extension, abduction-adduction and inward-outward rotation). RoMs in each joint were calculated for squats, gait and stair walking. For gait and stair walking, stride frequency (number of strides per minute) and two indices describing the overall pathology were derived. The indices were the GPS and the GDI* [33]. GPS summarizes the deviation of an individual’s joint angle curve from the mean joint angle curve of a control group on a point-to-point basis, while GDI* is a log transformed and scaled version of the GPS score.

### 2.4. Statistics

The free statistical software package R (version 3.5.3) was used for statistical analyses. Analysis of variance (ANOVA) was used to study group differences in demographic variables (age, height, weight, body mass index (BMI)). If group differences were significant, post-hoc tests were performed. A Levene’s test was performed to investigate the distribution homogeneity of the group data. No significant differences were found and hence a Tukey HSD was used for all post-hoc analyses. 

Linear mixed model designs were used to analyze group and side differences in kinematic outcome measures for each task separately (squat, gait, stair ascent, stair descent). For group comparisons, data from the non-dominant control legs and operated THAC/LFH legs were compared in a mixed model design with “Group” (THAC, LFH, CTRL) set as a fixed factor and “Subject” set as a random factor. For side comparisons within THAC and LFH groups, a mixed model design was used with “Group” (THAC, LFH), “Side” (Affected, Unaffected) and the interaction “Group × Side” set as fixed factors and “Subject” set as a random factor. The *p*-values were Bonferroni corrected and 95% confidence intervals of estimated marginal means are reported. Adjusted Bonferroni post-hoc analyses were used to assess pair-wise effects for significant factors and interactions. The significance level alpha was set as 0.05 for all statistical analyses.

## 3. Results 

**Figure 1:**
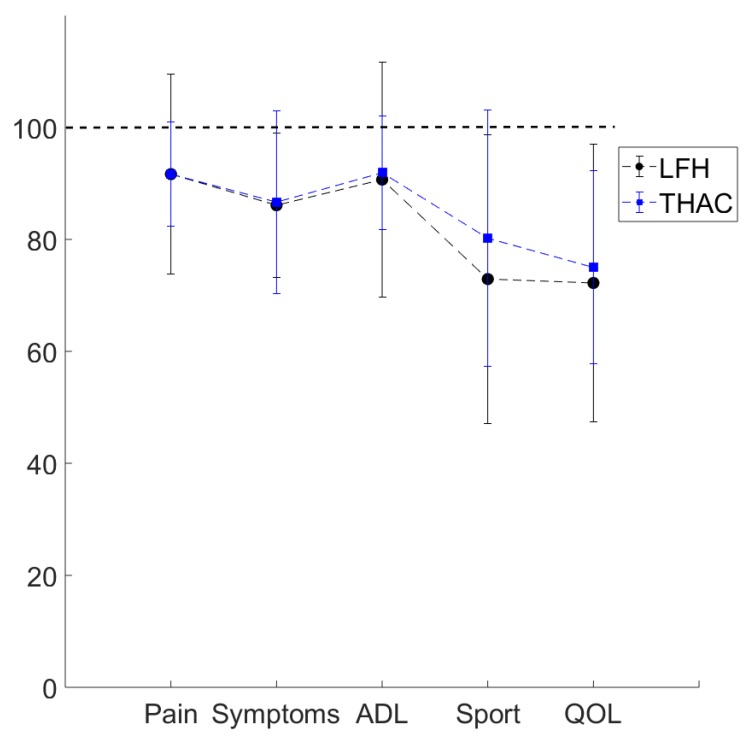
Hip dysfunction and Osteoarthritis Outcome Score (HOOS) profiles for the conventional THA prosthesis group (THAC) and large femoral head (LFH) prosthesis group. 100 indicates normal function and 0 indicates severe problems related to hip function. The five categories analyzed were Pain, Symptoms, Activities of daily living (ADL), Sport and recreation function (Sport) and Hip-related quality of life (QOL).

**Figure 2:**
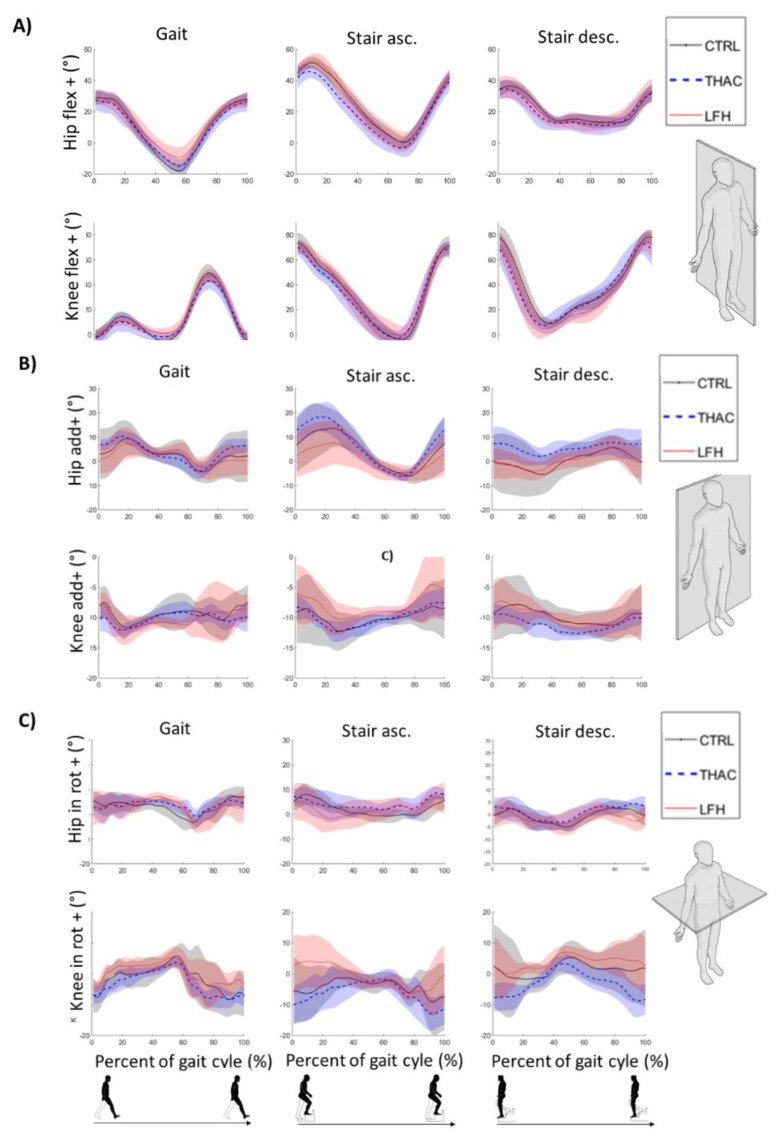
Angle curves in the hip and knee joints during gait and stair walking for sagittal plane motion (**A**), frontal plane motion (**B**) and transverse plane motion (**C**). The average angle curves are plotted with standard deviations as a shaded area for the non-dominant side of healthy controls (CTRL) and the operated side of the group with a conventional prosthesis (THAC) or large femoral head (LFH) design.

The HOOS profiles were lower than the reference values in both THAC and LFH groups (LFH: 83 ± 13, THAC: 84±19 groups, vs. normal function 100; see Figure 1). The motion curves in Figure 2 illustrate similar average angle curves in the hip joint in all groups during self-paced gait and stair walking, as reflected in the GDI* and GPS values that did not differ significantly between groups (Table 2). 

### 3.1. Squats

**Figure 3:**
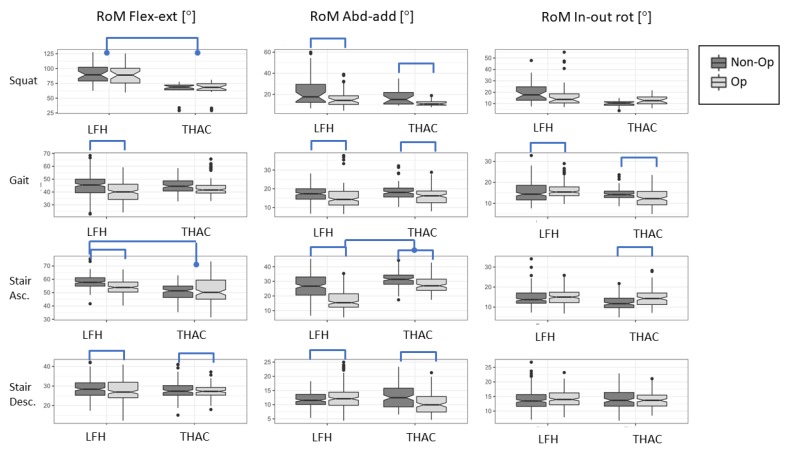
The boxplots illustrate range of motion (RoM) in the operated hips (light grey) and non-operated hips (dark grey) within the group with a total hip replacement (THAC) and the group with resurfaced hip design (LFH). Significant differences are marked in each graph, based on the statistical tests, between groups (brackets with end points) and between sides (simple brackets).

The LFH group had on average 27.2° significantly greater hip flexion-extension RoM compared to the THAC group (Table 3). They also had 5.2° significantly greater pelvic rotation ROM, 7.6° greater knee abduction-adduction ROM and 7.2° greater knee rotation RoM compared to the THAC group (Table 3). Side differences were evaluated in the patient groups (Table 4). Despite this task being performed with both legs simultaneously, the operated side had a significantly smaller hip abduction-adduction RoM and consequently smaller knee abduction-adduction for the THAC group, and a greater knee rotation RoM for both groups compared to the non-operated side (Table 4, Figure 3).

### 3.2. Gait

On average the LFH group had ~9° significantly smaller hip flexion-extension RoM during gait compared to controls (Table 3). Side asymmetries with smaller hip and knee ROM in the operated leg compared to the non-operated were found in flexion-extension (LFH), abduction-adduction (LFH in hip only; THAC in both hip and knee) and rotation (LFH in knee only; THAC in both hip and knee), see Table 4.

### 3.3. Stair Walking

During stair ascending, the LFH group had approximately 9° significantly smaller hip abduction-adduction RoM compared to both CTRL and THAC groups (Table 3). Side asymmetries during stair ascending were comparable to gait with smaller hip and knee RoM in the operated leg compared to the non-operated in flexion-extension (LFH) and abduction-adduction (both groups). In contrast to gait, THAC had greater hip rotation in the operated leg compared to the non-operated and both groups had greater knee rotation in the operated leg (see Table 4 and Figure 3).

When descending, the THAC group had 3.9° significantly smaller pelvic rotation RoM compared to controls (Table 3). Both groups had significantly smaller hip flexion-extension RoM in the operated leg compared to the non-operated leg (THAC: Non-op 27.9 (1.5) vs. Op 27.1 (1.5); LFH: Non-op 28.6 (1.3) vs. Op 27.4 (1.3); Table 4). In contrast to the LFH group, the THAC also had smaller hip abduction-adduction RoM in the operated leg compared to the non-operated leg, and instead greater knee abduction-adduction and rotation RoM in the operated leg compared to the non-operated leg.

## 4. Discussion

In this post-operative study, a wearable IMU-based movement analysis system was used to analyze whether femoral head size in hip arthroplasty influences movement patterns during squats, gait and stair walking. Motion parameters of clinical interest (RoM of lower body joint angles, GDI* and GPS) were analyzed in a group of asymptomatic controls, and two groups treated with different prosthesis designs; THAC or LFH.

### 4.1. Hip Function in Large Femoral Head (LFH) and Conventional Total Hip Arthroplasty (THAC) Designs: Side Asymmetries and Comparison to Controls

Our hypothesis was that the LFH design with the larger femoral head would allow a greater RoM, which was confirmed during squats where the LFH had on average 27° greater hip flexion RoM compared to the THAC group. Gait was investigated since this is central for independence and requires acceptable hip function. We expected a larger side asymmetry in gait in the THAC group when compared to the LFH group. However, the LFH group had a smaller hip flexion-extension RoM compared to controls and to the non-operated side (Table 3 and Table 4), while the THAC group did not. Both the THAC group and the LFH group had a larger hip abduction-adduction RoM on the operated side compared to their non-operated side. Furthermore, stair walking was investigated in order to study hip function during an everyday activity that is more strenuous than normal gait. We expected a larger side asymmetry in the THAC group when compared to the LFH group. Indeed, during stair descending, side differences in hip and knee RoMs were more pronounced in the THAC group (Table 4). During stair ascending, LFH had a larger hip flexion-extension RoM compared to the THAC group, but also a smaller hip abduction-adduction RoM compared to the THAC and CTRL groups (Table 3 and Table 4, Figure 3). In summary, hip function was still not fully recovered in either of the operated groups, instead both groups tended to load the non-operated leg to a higher extent than the operated leg. However, the LFH group had greater hip flexion-extension RoM compared to the THAC group during squats, when the operated leg was supported with the non-operated.

The GDI* and GPS scores were calculated to analyze movement quality during gait and stair walking. Rosenlund et al. [42], showed that the GDI* score during gait correlates to HOOS and hip strength, and we hence expected lower GDI* and GPS scores in the operated groups. Contrarily, our study did not show any significant group differences in GDI* and GPS between our operated groups and asymptomatic controls. It is possible that since GPS and GDI* are both constructed from a summarized deviation from all investigated joints, a significant group difference in a specific joint’s RoM may be too small to produce a significantly lower index. Hence, even though such indices are interesting to use from a clinical perspective, they should be combined with more detailed measures (e.g., RoM in single joints) to provide a more complete analysis of movement function.

### 4.2. Potential of Portable Movement Analysis Systems for Clinical Applications

In many current clinical evaluations of neurological or musculoskeletal disorders, movement function is assessed by clinical scales such as HOOS for hip function [15] or the Tinetti scale to evaluate gait [43]. Such tests allow identification of advanced alterations, but lack sensitivity to detect subtle changes. Movement analysis is an effective way of identifying subtle functional limitations, and analyses of gait and stair walking have proven to be valuable in the treatment and rehabilitation of neurological and musculoskeletal disorders [44,45]. Wearable IMU systems enable objective movement analysis outside the movement laboratory and have been shown to be reliable for clinical gait analysis [31]. Differences between the two systems originate mainly from sensor drift and skeleton model differences, see e.g., [32]. Since clinical examinations focus on short movement registrations (commonly a set of movement registrations where each registration is shorter than 5 minutes), the drift problem will have a relatively small impact. Inclusion of magnetometers further minimizes such drift, but it is important to remember that they are sensitive to ferromagnetic materials in the surrounding environment. Model differences may cause systematic differences in joint angle calculations, since optical camera-based laboratories most often use anatomical markers to define segment coordinate systems, while IMU systems instead use functional calibration. Standardization of protocol, sensor placement and gait variables is therefore important [46,47]. A well-known error source when analyzing body movement with either 3D optical camera systems or IMU-based systems is soft tissue artefacts from skin and muscle movements relative to the underlying bone, which may cause angular errors of more than 10 degrees [19,48,49]. Even though such errors can be minimized by careful placement of markers or sensors [50], they should be considered when evaluating the movement function with these methods.

### 4.3. Strengths and Limitations of the Current Study

A movement analysis system needs to be practical and user-friendly, with adequate reliability, for successful application in a clinical setting. The current system has been validated against a gold standard gait laboratory in a previous study and been shown to have a precision and accuracy within ~2–3° in comparison to this method for lower body joint angles during gait [40]. Even though methodological errors exist in both gold standard and portable IMU systems (e.g., due to skin movements [18,19]), random errors will cancel as the number of observations increase. In this study, the number of observations varies between 92 and 600, which we consider are large enough to ensure that the methodological error does not affect the results’ significance on a group level. 

The small group sizes of the current study could have affected the reported outcomes, particularly regarding the non-significant GDI* and GPS values. Despite the small group sizes, the results showed significant findings in line with our hypothesis, indicating a larger hip RoM in the LFH group and deviations from normal in both the LFH and THAC groups. A limitation was that the groups were matched regarding age, but not regarding weight or BMI. The THAC and LFH groups had significantly greater weight and BMI compared to controls. It is common that these patient groups have higher BMI since the hip osteoarthritis itself often leads to lower physical activity levels (as corroborated by a HOOS-Sports/recreation score of ~77 out of 100 for both of these groups). Since excess body weight may influence gait patterns [51], this may have contributed to the differences seen for lower body kinematics between our groups.

## 5. Conclusions

This study indicates that a hip prosthesis with an increased femoral head size results in greater hip flexion-extension RoM during squats and a smaller hip abduction-adduction RoM during stair ascending compared to the conventional design. However, side differences existed in both groups, which indicates that movement function was not fully recovered in either group. Wearable IMU-based systems could be used to identify subtle functional limitations after hip surgery and have the potential to be used for clinical evaluation of lower body function as an adjunct to clinical scales. The protocol and the selected parameters should, however, be evaluated in larger clinical studies.

## Figures and Tables

**Table 1:** Demographic data, years since operation and prosthesis head size given as mean ± standard deviation within parentheses for the conventional total hip replacement group (THAC), the large femoral head (LFH) design group and the control group (CTRL). Post hoc tests were performed if the analysis of variance (ANOVA) gave a significant group difference and are displayed as *p*-values in the last column. N = number of participants.

	THAC (N = 6)	LFH (N = 9)	Control (N = 8)	ANOVA (*p* Value)	Post hoc (*p* Value)
Age (year)	56 ± 9	49 ± 9	45 ± 12	0.370	
Weight (kg)	93 ± 12	90 ± 11	76 ± 7	0.009	LFH vs. THAC: 1.00 LFH > Control: 0.036 THAC > Control: 0.017
Height (m)	1.80 ± 0.07	1.82 ± 0.06	1.81 ± 0.05	0.803	
Body Mass Index	28 ± 2	27 ± 4	23 ± 2	0.010	LFH vs. THAC: 0.922 LFH vs. Control: 0.055 THAC > Control: 0.013
Years since operation	3.2 ± 1.2	4.7 ± 1.1	n/a	0.055	
Leg dominance; Right/Left	6/0	8/1	6/2	0.462	
Prosthesis head; diameter (mm) Range within brackets	32.7 (1.6) [32–36]	53.5 (3.2) [49–57]	-	0.000	

**Table 2:** Group estimated means and standard error (SE) in stride frequency, Gait Profile Score (GPS) and Gait Deviation Index (GDI*) for healthy controls (CTRL), the conventional total hip replacement group (THAC), and the large femoral head (LFH) design group. “*Num”* = number of observations (i.e., 23 participants x 5 trials x 4 cycles). The estimated group differences written in *cursive text*. “NS” = Non-significant.

Task	Group	Strides per Minute Mean (SE)	GPS Mean (SE)	GDI* Mean (SE)
Gait (*num* = 460)	CTRL THAC LFH	51.1 (2.8) 46.0 (3.3) 47.9 (2.7)	6.7 (0.4) 5.9 (0.5) 7.0 (0.4)	87.7 (1.1) 89.7 (1.2) 86.9 (1.0)
	*CTRL-THAC*	*−5.1 (4.3)*	*−0.9 (0.6)*	*2.0 (1.6)*
	*CTRL-LFH*	*−3.2 (3.9)*	*0.2 (0.6)*	*−0.8 (1.5)*
	Fixed effect	NS	NS	NS
Stair ascending (*num* = 460)	CTRL THAC LFH	53.0 (3.4) 52.5 (4.0) 53.0 (3.4)	7.4 (0.5) 7.7 (0.6) 7.4 (0.5)	87.9 (1.0) 86.8 (1.2) 85.9 (1.0)
	*CTRL-THAC*	*−0.4 (5.1)*	*0.4 (0.8)*	*−1.8 (1.5)*
	*CTRL-LFH*	*−7.8 (4.7)*	*0.9 (0.7)*	*−2.0 (1.4)*
	Fixed effect	NS	NS	NS
Stair descending (*num* = 460)	CTRL THAC LFH	61.9 (3.4) 55.4 (4.0) 52.9 (3.3)	7.1(0.4) 7.5 (0.5) 7.3 (0.4)	87.8(1.0) 86.5 (1.1) 87.2 (0.9)
	*CTRL-THAC*	*−6.5 (5.2)*	*0.4 (0.7)*	*−1.3 (1.5)*
	*CTRL-LFH*	*−9.0 (4.8)*	*0.2 (0.6)*	*−0.6 (1.3)*
	Fixed effect	NS	NS	NS

**Table 3:** Group estimated mean and standard error (SE) of the range of motion (RoM) for the healthy controls (CTRL), the conventional hip replacement group (THAC) and the large femoral head (LFH) design group. “FE” = flexion-extension; “Abd-Add” = abduction-adduction; “Rot” = inward-outward rotation and “*num*” = number of observations (i.e., squat 23 participants x 4 cycles; gait and stair walking 23 participants × 5 trials × 4 cycles). The estimated group differences are given in *cursive* text. Post hoc tests were performed if a significant group difference was found.

Task	Group	RoM Pelvis (°)			RoM Hip (°)			RoM Knee (°)		
		FE	Abd-Add	Rot	FE	Abd-Add	Rot	FE	AbAdd	Rot
**Squat** (*num* = 92)	CTRL THAC LFH	24.5 (3.9) 16.8 (4.5) 28.1 (3.7)	4.6 (1.8) 4.7 (2.0) 9.6 (1.7)	4.4 (1.2) 3.7 (1.4) 8.9 (1.2)	79.4 (6.8) 63.6 (7.8) 91.0 (6.4)	13.3 (2.6) 11.9 (2.9) 17.0 (2.4)	9.9 (2.9) 13.2 (3.4) 18.0 (2.8)	90.5 (5.3) 80.9 (6.2) 95.7 (5.0)	13.2 (1.9) 9.2 (2.2) 16.8 (1.8)	15.2 (1.8) 12.2 (2.1) 19.4 (1.7)
	*CTRL-THAC*	*−7.7 (6.0)*	*0.1 (2.7)*	*−0.7 (1.9)*	*−15.8 (10.3)*	*−1.4 (3.9)*	*3.3 (4.5)*	*−9.6 (8.1)*	*−4.0 (2.9)*	*−3.1 (2.8)*
	*CTRL-LFH*	*3.6 (5.4)*	*5.0 (2.4)*	*4.5 (1.7)*	*11.6 (9.3)*	*3.8 (3.5)*	*8.1 (4.0)*	*5.2 (7.3)*	*3.6 (2.7)*	*4.1 (2.5)*
	Fixed effects	NS	NS	*p* = 0.014	*p* = 0.043	NS	NS	NS	*p* = 0.050	*p* = 0.046
	Post hoc			LFH>THAC LFH>CTRL	LFH>THAC				LFH>THAC	LFH>THAC
**Gait** (*num* = 460)	CTRL THAC LFH	5.7 (0.4) 6.6 (0.5) 6.4 (0.4)	7.1 (0.7) 6.9 (0.8) 7.3 (0.7)	11.9 (1.0) 9.8 (1.2) 9.8 (1.0)	49.9 (2.5) 43.9 (2.9) 40.5 (2.4)	19.7 (1.6) 16.0 (1.9) 15.2 (1.5)	16.9 (1.4) 12.9 (1.6) 16.2 (1.3)	57.3 (1.8) 50.1 (2.1) 51.7 (1.7)	16.0 (1.2) 12.0 (1.4) 15.2 (1.1)	19.1 (1.2) 16.0 (1.4) 15.7 (1.1)
	*CTRL-THAC*	*0.9 (0.7)*	*−0.2 (1.1)*	*−2.2 (1.6)*	*−6.0 (3.8)*	*−3.7 (2.4)*	*−4.0 (2.1)*	*7.1 (2.7)*	*4.0 (1.8)*	*−3.1 (1.8)*
	*CTRL-LFH*	*0.7 (0.6)*	*0.2 (0.9)*	*−2.1 (1.4)*	*−9.4 (3.5)*	*−4.5 (2.2)*	*−0.7 (2.0)*	*−5.6 (2.5)*	*−0.8 (1.7)*	*−3.3 (1.6)*
	Fixed effects	NS	NS	NS	*p* = 0.044	NS	NS	*p* = 0.037	NS	NS
	Post hoc				CTRL>LFH			*No sig. post hoc effects*		
**Stair Ascending** (*num* = 460)	CTRL THAC LFH	7.0 (0.7) 7.8 (0.8) 7.0 (0.7)	12.8 (1.2) 15.8 (1.4) 14.1 (1.1)	9.4 (1.1) 6.1 (1.2) 7.4 (1.0)	53.4 (1.8) 51.7 (2.1) 54.1 (1.7)	26.1 (1.9) 28.0 (2.2) 17.4 (1.81)	12.1 (1.1) 14.5 (1.2) 14.9 (1.0	79.3 (1.8) 77.3 (2.1) 75.9 (1.7)	13.9 (1.6) 13.8 (1.8) 17.5 (1.5)	16.3 (1.8) 18.5 (2.1) 20.3 (1.7)
	*CTRL-THAC*	*0.8 (1.1)*	*3.0 (1.8)*	*−3.2 (1.6)*	*−1.7 (2.8)*	*1.9 (2.9)*	*2.4 (1.6)*	*−2.0 (2.7)*	*−0.1 (2.4)*	*2.2 (2.7)*
	*CTRL-LFH*	*0.1 (1.0)*	*1.2 (1.7)*	*−2.0 (1.5)*	*0.7 (2.5)*	*−8.8 (2.7)*	*2.8 (1.4)*	*−3.4 (2.5)*	*3.6 (2.1)*	*4.0 (2.5)*
	Fixed effects	NS	NS	NS	NS	*p* = 0.002	NS	NS	NS	NS
	Post-hoc					CTRL> LFH THAC> LFH				
**Stair Descending** (*num* = 460)	CTRL THAC LFH	6.4 (0.4) 6.4 (0.5) 6.2 (0.4)	8.3 (0.9) 8.4 (1.1) 8.7 (0.9)	10.2 (0.8) 6.3 (1.0) 7.3 (0.8)	29.1 (1.3) 27.1 (1.6) 27.4 (1.3)	12.8 (1.0) 10.6 (1.1) 12.6 (0.9)	14.7 (0.7) 13.9 (0.8) 14.3 (0.7)	74.4 (1.4) 73.3 (1.7) 75.1 (1.4)	11.4 (1.2) 11.4 (1.3) 14.1 (1.1)	16.4 (1.2) 18.2 (1.4) 17.0 (1.2)
	*CTRL-THAC*	*−0.0 (0.6)*	*−0.1 (1.4)*	*−3.9 (1.3)*	*−2.1 (2.1)*	*−2.2 (1.5)*	*−0.8 (1.1)*	*−1.1 (2.2)*	*−0.0 (1.9)*	*1.9 (1.8)*
	*CTRL-LFH*	*−0.2 (0.6)*	*0.4 (1.3)*	*−2.9 (1.1)*	*−1.7 (1.9)*	*−0.2 (1.3)*	*−0.4 (1.0)*	*0.7 (2.0)*	*2.7 (1.6)*	*0.6 (1.7)*
	Fixed effects	NS	NS	*p* = 0.013	NS	NS	NS	NS	NS	NS
	Post hoc			CTRL>THAC						

**Table 4:** Adjusted marginal means (Mean) and standard errors (SE) for range of motion (RoM) in non-operated and operated legs in the conventional hip replacement group (THAC) and the large femoral head (LFH) design group. “FE” = Flexion-extension; “Abd-Add” = Abduction-adduction; “Rot” = inward-outward rotation and “*num*” = the number of observations (Squat: 15 participants × 4 cycles × 2 legs; Gait and Stair ascending/descending: 15 participants x 5 trials × 4 cycles × 2 legs). The significance level *p* is reported for significant fixed effects and interactions (Group, Side, Group×Side); “NS” = non-significance. Post hoc tests were performed if a significant interaction was found. The notation “$” denotes misleading significance due to involvement in interactions.

Task	RoM	Direction	THAC Mean (SE)		LFH Mean (SE)		Fixed Effects (*p* Value)			Significant group and side difference specified. *Post hoc tests given instead if Grp* *× Side is significant.*
			Non-op	Op	Non-op	Op	Group	Side	Grp× Side	
**Squat** (*num* = 120)	Pelvis (°)	FE	16.8 (3.8)	16.8 (3.8)	28.1 (3.2)	28.1 (3.2)	0.005	NS	NS	**Group:** THAC<LFH
		Abd-Add	4.7 (2.5)	4.7 (2.5)	9.6 (2.0)	9.6 (2.0)	NS	NS	NS	
		Rot	3.7 (1.7)	3.7 (1.7)	8.9 (1.4)	8.9 (1.4)	0.036	NS	NS	**Group:** THAC<LFH
	Hip (°)	FE	63.2 (7.0)	63.6 (7.0)	91.0 (5.8)	91.0 (5.8)	0.010	NS	NS	**Group:** THAC<LFH
		Abd-Add	17.4 (3.6)	11.9 (3.6)	22.8 (3.1)	17.0 (3.1)	NS	0.000	NS	**Side:** Non-op > Op
		Rot	9.9 (3.3)	13.2 (3.3)	20.0 (2.7)	18.0 (2.7)	NS	NS	0.002	*Post hoc tests not significant*
	Knee (°)	FE	80.4 (6.2)	80.9 (6.2)	97.8 (4.9)	95.7 (4.9)	NS	NS	NS	
		Abd-Add	13.8 (1.9)	9.2 (1.9)	15.3 (1.5)	16.8 (1.5)	NS	NS	0.001	***THAC:*** *Non-op > Op,* ***Op:*** *LFH>THAC*
		Rot	14.6 (2.7)	12.2 (2.7)	21.8 (2.2)	19.4 (2.2)	NS	0.008	NS	**Side:** Non-op > Op
**Gait** *(num = 600)*	Pelvis (°)	FE	6.9 (0.4)	6.6 (0.4)	6.4 (0.4)	6.4 (0.4)	NS	NS	NS	
		Abd-Add	7.0 (0.7)	6.9 (0.7)	7.5 (0.6)	7.3 (0.6)	NS	NS	NS	
		Rot	10.2 (1.3)	10.2 (1.3)	9.9 (1.2)	9.8 (1.2)	NS	NS	NS	
	Hip (°)	FE	45.2 (2.6)	43.9 (2.6)	44.8 (2.4)	40.5 (2.4)	NS	0.000^$^	0.000	***LFH:*** *Non-op > Op*
		Abd-Add	17.4 (1.1)	16.0 (1.1)	17.0 (1.0)	15.2 (1.0)	NS	0.000	NS	**Side:** Non-op > Op
		Rot	15.9 (1.2)	13.3 (1.2)	15.3 (1.0)	16.2 (1.0)	NS	NS	0.000	***THAC:*** *Non-op > Op* ***LFH:*** *Non-op < Op*
	Knee (°)	FE	49.3 (1.8)	48.4 (1.8)	54.6 (1.4)	51.7 (1.4)	NS	0.000^$^	0.002	***LFH:*** *Non-op>Op*
		Abd-Add	13.7 (1.0)	12.2 (1.0)	13.5 (0.9)	15.2 (0.9)	NS	NS	0.000	***THAC:*** *Non-op > Op* ***LFH:*** *Non-op < Op*
		Rot	19.5 (1.2)	16.0 (1.2)	18.1 (1.1)	15.7 (1.1)	NS	0.000^$^	NS	**Side:** Non-op > Op
**Task**	**Range of Motion**	**Direction**	**THAC** **Non-op**	**THAC** **Op**	**LFH** **Non-op**	**LFH** **Op**	**Group**	**Side**	**Grp × Side**	
**Stair Ascending** *(num = 600)*	RoM Pelvis (°)	FE	8.0 (0.8)	7.8 (0.8)	6.5 (0.6)	7.0 (0.6)	NS	NS	0.027*	***LFH:*** *Non-op < Op*
		Abd-Add	16.2 (1.5)	15.8 (1.5)	13.9 (1.2)	14.1 (1.2)	NS	NS	NS	
		Rot	6.3 (1.0)	6.1 (1.0)	7.2 (0.8)	7.4 (0.8)	NS	NS	NS	
	RoM Hip (°)	FE	50.9 (1.3)	51.7 (1.3)	58.1 (1.0)	54.1 (1.0)	0.009^$^	0.000^$^	0.000	***LFH*** ***:*** *Non-op > Op* *LFH Non-Op > THAC Op* *LFH Non-Op > THAC Non-Op*
		Abd-Add	31.3 (2.0)	28.0 (2.1)	26.3 (1.7)	17.4 (1.7)	0.010^$^	0.000^$^	0.000	***LFH*** ***, THAC:*** *Non-Op>Op* *THAC Non-Op > LFH Op* *THAC Op > LFH Op*
		Rot	12.2 (0.9)	14.5 (0.9)	14.7 (0.8)	14.9 (0.8)	NS	0.000^$^	0.000	***THAC:*** *Non-op < Op*
	RoM Knee (°)	FE	76.3 (1.9)	77.3 (1.9)	78.4 (1.6)	75.9 (1.6)	NS	0.005^$^	0.000	***LFH:*** *Non-op > Op*
		Abd-Add	15.3 (1.6)	13.8 (1.6)	19.6 (1.3)	17.5 (1.3)	NS	0.000	NS	**Side:** Non-Op > Op
		Rot	17.2 (1.5)	18.5 (1.5)	17.8 (1.2)	20.3 (1.2)	NS	0.000	NS	**Side:** Non-Op < Op
**Stair Descending** *(num = 600)*	RoM Pelvis (°)	FE	6.1 (0.6)	6.4 (0.6)	6.3 (0.5)	6.2 (0.5)	NS	NS	NS	
		Abd-Add	8.8 (1.0)	8.4 (1.0)	8.5 (0.9)	8.7 (0.9)	NS	NS	NS	
		Rot	6.3 (0.8)	6.3 (0.8)	7.2 (0.6)	7.3 (0.6)	NS	NS	NS	
	RoM Hip (°)	FE	27.9 (1.5)	27.1 (1.5)	28.6 (1.3)	27.4 (1.3)	NS	0.000	NS	**Side:** Non-op > Op
		Abd-Add	12.6 (1.0)	10.6 (1.0)	11.8 (0.8)	12.6 (0.8)	NS	NS	0.000	***THAC:*** *Non-op > Op* ***LFH:*** *Non-op < Op*
		Rot	14.0 (0.9)	13.9 (0.9)	14.0 (0.7)	14.3 (0.7)	NS	NS	NS	
	RoM Knee (°)	FE	70.5 (1.5)	73.3 (1.5)	74.5 (1.2)	75.1(1.2)	NS	0.000^$^	0.002	***THAC:*** *Non-Op < Op*
		Abd-Add	14.3 (1.4)	11.4 (1.3)	14.2 (1.0)	14.1 (1.0)	NS	0.000^$^	0.000	***THAC:*** *Non-op > Op*
		Rot	21.5 (1.4)	18.2 (1.5)	17.9 (1.2)	17.0 (1.2)	NS	0.000^$^	0.001	***THAC:*** *Non-Op > Op*
